# Students’ engagement with AI-supported learning and its association with academic interest and career intentions in business analytics education

**DOI:** 10.1371/journal.pone.0350661

**Published:** 2026-06-04

**Authors:** Yang Cheng, Jaekuk Lee, Florence Martin, William Rand

**Affiliations:** 1 Department of Communication, North Carolina State University, Raleigh, North Carolina, United States of America; 2 CRDM Program, North Carolina State University, Raleigh, North Carolina, United States of America; 3 Teacher Education and Learning Sciences, College of Education, North Carolina State University, Raleigh, North Carolina, United States of America; 4 Department of Marketing, Poole College of Management, North Carolina State University, Raleigh, North Carolina, United States of America; MCC Boyd Tandon School of Business, INDIA

## Abstract

Artificial intelligence (AI) tools are increasingly embedded in higher education, yet limited research has examined how sustained AI usage intentions in AI-supported learning environments are associated with learning motivation and longer-term educational development. Treating AI use as a course-embedded learning experience rather than a discrete adoption decision, this study investigates how students’ perceptions of AI-supported learning are associated with continued usage intentions and how such intentions subsequently relate to academic interest and career-related intentions. Grounded in post-adoption technology continuance research, motivation theory, and Social Cognitive Career Theory, we develop and test a structural model linking perceived AI enhancement, interactivity, fun, and coolness to continued AI usage intentions, academic interest, and career-choice intentions. Survey data were collected from undergraduate students enrolled in business analytics courses and analyzed using structural equation modeling. The results show that perceived enhancement, interaction, fun, and coolness are each significantly associated with continued AI usage intentions in coursework. Continued AI usage intentions, in turn, are positively related to academic interest in business analytics, and academic interest statistically mediates the relationship between continued AI usage intentions and career-choice intentions. However, indirect effects from these antecedent variables to academic interest through continued AI usage intentions were not statistically significant. By conceptualizing continued AI usage intentions as an ongoing learning process that is linked to how students engage with disciplinary knowledge over time, this study advances understanding of the developmental role of AI-supported instruction in higher education. The findings contribute to research on technology, knowledge, and learning, and offer practical implications for designing AI-supported learning environments that foster sustained usage intentions, interest development, and future-oriented educational pathways.

## Introduction

Artificial intelligence (AI) has rapidly transitioned from experimental use to routine practice in higher education, reshaping how students engage with learning tasks, information resources, and academic support. Large-scale survey research documents widespread and sustained use of generative AI tools, such as ChatGPT, for information seeking, writing assistance, and coursework support across diverse student populations [1,2]. Longitudinal evidence further indicates that students’ beliefs and perceptions of these tools are key predictors of continued usage intentions over time, signaling a shift from initial exploration to habitual academic engagement [3]. Complementing these findings, systematic and meta-systematic reviews report substantial growth in AI-in-education research and practice, with applications ranging from adaptive tutoring and automated feedback to personalized learning support. Collectively, this literature suggests that AI technologies are becoming embedded features of higher education learning environments, rather than short-lived technological novelties [4–6].

Research on post-adoption technology use provides important insights into why learners continue engaging with educational technologies beyond initial exposure and consistently highlights the role of both utilitarian and affective factors. The Expectation-Confirmation Model of information systems continuance identifies post-use satisfaction and perceived usefulness as primary drivers of continuance intention [7]. Related frameworks further demonstrate that performance expectancy, effort expectancy, hedonic motivation, social influence, and habit contribute meaningfully to sustained use [8,9]. Within educational contexts, empirical studies similarly show that confirmation, satisfaction, usefulness, and enjoyment predict continued usage with learning technologies [10,11]. Applied to AI-supported learning, these determinants correspond to students’ perceptions of system design and interaction quality.

Enhancement and interaction reflect instrumental and experiential features that support effective learning engagement, whereas fun and the social appeal captured by coolness reflect affective pathways associated with enjoyment and hedonic motivation. However, much of the post-adoption literature remains focused on explaining repeated use itself, offering limited insight into how sustained usage intentions with educational AI tools function as a learning experience that is associated with broader motivational and developmental outcomes.

Social Cognitive Career Theory (SCCT) offers a useful framework for extending continuance research by conceptualizing sustained usage intentions as a learning experience associated with interest development and future-oriented intentions. Within SCCT, learning experiences are related to self-efficacy beliefs and outcome expectations, which foster domain interest; this interest subsequently guides choice goals and career-relevant intentions and behaviors [12]. Meta-analytic evidence provides robust support for these pathways across domains, demonstrating that self-efficacy and outcome expectations predict interests, and that interests, in turn, predict choice goals, with contextual support and barriers operating largely through these cognitive mechanisms [13]. More recent reviews further emphasize the central role of interest in guiding educational and career-related choices and highlight how learning environments channel the motivational impact of experiences over time [14]. Complementary research on interest development similarly shows that engaging and meaningful learning experiences can trigger and sustain interest, supporting attention, persistence, and re-engagement in academic domains [15,16]. From this perspective, sustained use of AI tools in coursework may function as a formative, knowledge-mediated learning experience associated with the development of academic interest and subsequent career-related intentions. While self-efficacy and outcome expectations are central components of SCCT, the present study focuses specifically on the interest–choice pathway and does not include these constructs in the structural model, consistent with a parsimonious modeling approach and the study’s emphasis on AI-supported learning engagement.

Business analytics represents a particularly appropriate context for examining these processes because it integrates data analysis, decision-making, and applied technology skills that are central to contemporary business education. Analyses of large-scale job postings and employer surveys consistently identify strong demand for analytics and data science competencies alongside communication, visualization, and problem-solving skills [17–19]. Program-level studies in business and information systems education similarly examine alignment between curricula and labor market expectations and frequently report an emphasis on technical skills accompanied by gaps in applied and transferable competencies valued by employers [20,21]. This literature positions business analytics courses that integrate AI-supported tools as a meaningful educational setting for examining how students’ perceptions of AI-supported learning relate to sustained usage intentions, interest development, and early career intentions. Students’ regular interaction with AI tools in such courses involves both instrumental benefits and experiential qualities that may be associated with continued usage intentions and longer-term motivational outcomes, yet empirical research directly testing these relationships remains limited.

Building on this context, the present study develops and tests a structural model linking students’ perceptions of AI-supported learning environments to continued usage intentions, academic interest, and career intentions in business analytics education. Perceptions of design-related qualities, operationalized as enhancement, interactivity, fun, and coolness, are modeled as antecedents of continued AI usage intentions, consistent with post-adoption research emphasizing usefulness, satisfaction, hedonic motivation, social influence, and habit as key drivers of continuance [7–9]. Continued AI usage intentions are conceptualized as ongoing learning-related orientations through which students engage with disciplinary knowledge, develop academic interest, and form career-related intentions, consistent with Social Cognitive Career Theory [12–14]. The framework also draws on research on AI-mediated communication, which highlights how intelligent systems are linked to user experiences and interaction processes in ways that are associated with engagement and learning [22]. Together, these perspectives motivate an empirical investigation using structural equation modeling and offer design-relevant insights for educators seeking to integrate AI into coursework in ways that support sustained usage intentions, interest formation, and developmental learning outcomes.

## Literature review

### Continued usage intentions with AI tools

Research on technology use distinguishes initial adoption from post-adoption continuance, noting that the determinants of trying a system and those of keeping it routine are related, yet not identical. The Expectation-Confirmation Model (ECM) explains this distinction by proposing that after initial experience, users compare what they expected with what occurred. When experience is confirming, it strengthens perceived usefulness and satisfaction, and those in turn drive continuance intention [7]. Studies in educational and training settings echo this logic and repeatedly show that usefulness and satisfaction remain central to decisions to continue beyond first exposure [10]. In short, continuance reflects how well a tool works for users in practice and whether the experience feels worth repeating.

Continuance is not purely utilitarian. For systems with mixed utilitarian-hedonic value, enjoyment can meaningfully bolster sustained use. Work on hedonic information systems demonstrates that perceived enjoyment can predict usage on par with usefulness when interactions are intrinsically rewarding [11]. Complementary theorizing on cognitive absorption explains how deep, enjoyable involvement characterized by focused immersion and temporal dissociation can reinforce beliefs that support ongoing use [23]. Generative AI assistants in academic settings combine productivity features such as summarizing, drafting, and debugging with interactive exploration and conversational engagement. They also enable interactive exploration that may be intrinsically engaging, underscoring the dual importance of usefulness and enjoyment.

Broader acceptance frameworks incorporate these experiential drivers into post-adoption behavior. Unified Theory of Acceptance and Use of Technology 2 (UTAUT2) extends expectancy beliefs with hedonic motivation and habit, emphasizing that repeated positive interactions can consolidate into routine, low-friction use, while enjoyment sustains engagement over time [9]. Habit helps explain persistence after novelty declines and clarifies why intention and behavior may diverge when use becomes automatic [8]. For clarity in this paper, continuance refers to the intention to keep using an AI tool as part of routine coursework, with the understanding that intention, reuse, and stickiness are related but analytically distinct constructs.

Another line of research highlights how features of technology are related to users’ perceptions and motivation. Studies on interactivity show that when tools respond predictably, allow user control, and support back-and-forth engagement, they can increase users’ confidence, satisfaction, and perceived effectiveness [24,25]. These principles help explain why repeated use of AI tools in coursework can sustain interest, even across different types of generative or analytical technologies. The MAIN framework further clarifies these dynamics by proposing that modality, agency, interactivity, and navigability serve as heuristic cues. Users may infer from these cues that a system is smart or novel, that they are in control, that the system responds to them, and that they can navigate content easily. These inferences can boost perceived effectiveness and enjoyment, which in turn encourage re-engagement [26]. In the context of generative AI, these cues are instantiated in concrete affordances: conversational responsiveness reflects Interactivity, perceived autonomy or initiative reflects Agency, and presentation formats that make outputs easy to apply reflect modality and navigability. Linking these affordances to the MAIN cues helps explain how repeated engagement with AI in coursework can reinforce perceptions of control, usefulness, and enjoyment, supporting sustained motivation and interest in the subject.

These traditions motivate a dual-path account of post-adoption engagement in which sustained use is anchored in functional enhancement and affective experience, often reinforced by habit. Building on this account, this study conceptualizes four design-relevant factors as key antecedents of AI continuance: (1) Coolness, (2) Fun, (3) Enhancement, and (4) Interaction. Coolness reflects the social and aesthetic appeal of AI tools, including qualities such as appropriate novelty, distinctiveness, or autonomy-implicating features. Research shows that coolness can motivate favorable evaluations and approach behaviors, and although it is not a classic ECM variable, it may be related to continuance by increasing affect and perceived value [27,28]. Fun captures affective enjoyment that arises from using AI tools, helping to sustain interest in mixed utilitarian-hedonic contexts. Perceived enhancement refers to students’ perceptions that AI-supported learning tools expand their ability to ask questions, express insights, and engage with learning content in personally meaningful ways. Interaction emphasizes experiential engagement, including responsiveness and controllability, which facilitate iterative prompt-and-feedback cycles and strengthen perceived efficacy and continued usage intentions. Together, these four factors represent complementary pathways: socio-aesthetic, affective, functional, and experiential that support students’ ongoing engagement with AI in coursework.

### Perceived coolness

Perceived coolness is an affective, multidimensional psychological judgment users make about technology, encompassing its positive, innovative, and aesthetically appealing qualities. It is a complex construct that includes dimensions such as originality, attractiveness, and subcultural appeal [29]. In today’s competitive technology landscape, where core features are often quickly replicated, coolness has emerged as a crucial differentiator for new and innovative products, including generative AI tools [30]. The perception of coolness moves beyond simple functionality (utilitarian value) to tap into users’ hedonic and social motivations.

Coolness acts as a powerful stimulus that reinforces a product’s affectively driven qualities. When a user perceives an AI tool to be cool, this perception enhances the positive emotional responses they have toward the product, such as feelings of excitement and delight [31]. This positive affective state directly translates into a favorable overall evaluation of the system experience. Studies examining innovative technologies, such as virtual reality and service robots, have repeatedly demonstrated that perceived coolness is a significant antecedent of positive outcomes, including intention to use and, most relevantly, learning motivation and learning satisfaction [32,33]. Consequently, this study expects that when students find the AI tools to be aesthetically appealing, novel, and technologically impressive, they will express higher levels of satisfaction with their educational experience. This leads to the following hypothesis.

H1a: Perceived coolness will be positively associated with continued AI usage intentions.

### Perceived fun

Perceived fun, often operationalized as hedonic motivation, is defined as the extent to which the activity of using a specific information system is perceived to be enjoyable in its own right, apart from any performance consequences that may result from its use [34]. Unlike utilitarian beliefs like perceived usefulness, which are extrinsic and focus on task performance, fun is an intrinsic motivator [35]. While early technology acceptance models focused primarily on utilitarian factors, the success of modern digital tools, particularly in educational and social contexts, has highlighted the critical role of enjoyment in driving adoption and sustained usage intentions [36,37]. In the context of generative AI, where users engage in creative tasks, dialogue, and exploration, the subjective experience of enjoyment becomes highly relevant. When a system provides a pleasurable and engaging experience, it is positively related to a user’s attitude toward the system, which in turn leads to higher satisfaction [38,39]. High levels of intrinsic motivation derived from fun activities encourage students to use the AI tool more frequently and for longer durations, thereby reinforcing their satisfaction with the learning outcomes and the tool itself [40]. Therefore, the fun derived from interacting with the AI is expected to be a direct and positive determinant of the user’s affective state in the post-adoption phase. This leads to the following hypothesis.

H1b: Perceived fun will be positively associated with continued AI usage intentions.

### Perceived enhancement

Perceived enhancement serves as the primary cognitive and utilitarian mechanism driving students’ intention to continue using AI tools. This construct is one of the most robust and widely validated predictors of technology adoption and sustained use across the entire information system literature [9,41]. Specifically, perceived enhancement refers to students’ beliefs that AI tools enhance their ability to ask questions, express their insights, and communicate their ideas in meaningful ways, thereby improving the effectiveness and overall quality of their learning and academic work [1]. Within a business analytics curriculum, enhancement manifests as the perceived ability of AI tools to help students explore analytical problems, communicate their ideas, and engage more actively with data-driven learning tasks. When students perceive that AI tools support these forms of inquiry, expression, and engagement, their beliefs in the value of the technology are reinforced.

Within the Expectation Confirmation Model (ECM), perceived enhancement operates as a revised belief about the technology’s utility formed after actual experience [7]. The process begins after initial adoption, followed by direct usage experience. During this period of use, the student assesses the tool’s actual performance against their initial expectations, a process known as confirmation. If the tool consistently yields academic benefits that meet or exceed these initial expectations, a state referred to as positive confirmation, students’ perception of its usefulness is strengthened and updated [7,42]. This enhanced cognitive belief then acts as a direct antecedent to both user satisfaction and, subsequently, long-term continuance intention. Thus, the utilitarian value provided by the AI tools is not a static measure, but a continually updated assessment based on confirmed performance gains.

The significance of perceived enhancement is amplified in professional programs requiring high analytic rigor, such as business analytics. In these contexts, AI tools can support students in asking questions, exploring analytical problems, expressing their insights, and articulating their perspectives during data-driven learning activities. Features such as code generation, query formulation, and information synthesis may facilitate inquiry and exploration by helping students engage more actively with complex course content. When these tools reliably automate complex yet routine tasks, they allow students to divert cognitive resources toward higher-order analytical thinking and critical problem solving, thereby accelerating the acquisition of expertise [43]. Empirical evidence from educational technology research supports this pathway, suggesting that technologies that facilitate meaningful engagement, learning, and problem solving are associated with stronger continued usage intentions in e-learning environments [10]. Given that AI tools can enhance students’ opportunities for inquiry, self-expression, and meaningful engagement with learning activities, this study expects perceived enhancement to be a significant driver of their long-term commitment to using the technology.

H1c: Perceived enhancement will be positively associated with AI usage intentions.

### Perceived interaction

Perceived interaction represents the user’s subjective experience and belief regarding the system’s ability to facilitate a responsive and reciprocal exchange [25]. In the context of generative AI tools, which are inherently dialogic, perceived interaction moves beyond simple functionality and becomes an important aspect of AI-mediated communication experiences [44]. It is defined not only by the technical presence of interactive features, but also by users’ perceptions of responsiveness and active engagement during communication with AI systems [45]. The construct of perceived interaction involves dimensions such as responsiveness, user control, and communication-related engagement [45–46]. As a critical external variable, perceived interaction acts as a powerful antecedent in continuance models, similar to the previously discussed perceived enhancement. When users perceive an AI tool to be highly interactive, meaning it provides timely feedback and allows for meaningful dialogue, they are more likely to generate favorable affective evaluations of their experience. This positive interaction directly leads to increased satisfaction with the learning outcome and the overall system use [37]. Furthermore, effective interaction leads to enjoyment, tapping into hedonic motivation, which is a powerful driving force of long-term engagement [47]. Consequently, perceived interaction is expected to be positively associated with user satisfaction and continued usage intentions. This leads to the following hypothesis.

H1d: Perceived interaction will be positively associated with continued AI usage intentions.

### Continued AI usage intentions and academic interest

Continued usage intention is expected to be related to motivation by reinforcing beliefs about capability and value during authentic coursework. Interest develops over time through a progression from triggered situational interest to maintained situational interest and then to emerging and well-developed individual interest. Repeated encounters that feel meaningful and effective help interest move along this trajectory, especially when students experience growing competence and relevance in the activity itself [16]. Expectancy-value theory aligns with this pathway by proposing that students invest in tasks they expect to succeed in and that they value, which implies that productive interactions with AI that clarify utility and support success should deepen and sustain interest across tasks and time [48]. Self-determination theory adds that contexts supporting competence and autonomy foster intrinsic motivation and interest, and iterative prompt and feedback cycles can provide those experiences when students feel effective and in control during coursework [49].

These motivation processes connect to longer-term development through social cognitive career theory. SCCT holds that learning experiences inform self-efficacy and outcome expectations, which are then linked to interests that guide later choices. Hands-on interactions that make AI tools usable and useful count as learning experiences that can strengthen academic interest in domains where those tools matter, and evidence across fields suggests that these mechanisms generalize widely [12,14]. Interest is most likely to deepen when engagement is productive and goal-congruent rather than merely frequent, which is consistent with research showing that interest can be promoted when learners experience progress on valued goals and see clear relevance to those goals [15]. Prior interest may also promote AI use, suggesting a potentially bidirectional relationship; however, this hypothesis emphasizes the pathway where sustained, effective engagement enhances interest [14]. Together, these motivational processes help explain how continued AI usage intentions may be associated with academic interest and subsequent career-choice intentions.

H2: Continued AI usage intentions will be positively associated with academic interest in business analytics.

### Academic interest and career-choice intentions

Academic interest is widely treated as a proximal driver of career-relevant intentions. Social cognitive career theory proposes a pathway in which learning experiences are related to self-efficacy and outcome expectations, those beliefs foster interests, and interests guide subsequent choice goals and intentions [12,14]. In the present framework, the implication is direct. As continued usage intentions of AI supports experiences that cultivate interest in business analytics, interest should function as a proximal antecedent of intentions to pursue study or work in that domain [12,14].

This paper uses career-choice intentions to refer to self-reported plans to pursue business analytics in education or employment rather than choice goals or enacted choices. Later sections operationalize this construct with a multi-item intention scale to avoid drift toward frequency or behavior. Meta-analytic evidence reinforces the interest-to-intention link. A synthesis across Holland themes reported reliable paths from interest to choice goals after accounting for self-efficacy and outcome expectations, indicating that interest contributes uniquely to choice formation [13]. Work focused on science and technology contexts reached a similar conclusion and showed that interest operates alongside efficacy and expectations to predict choice options in those fields [50]. These findings support the expectation that when students’ interest in business analytics strengthens, intentions to pursue related academic or occupational paths rise as well.

Prior interest can also encourage technology use, which makes the association plausibly reciprocal, yet the hypothesis centers on the interest-to-intention pathway documented in SCCT syntheses and meta-analyses [13,14].

H3: Academic interest in business analytics will be positively associated with career-choice intentions in business analytics.

### Direct effect of continued AI usage on career-choice intentions

Social cognitive career theory positions interest as a proximal driver of choice intentions, yet it also allows for direct influences on choice goals under specific conditions. Learning experiences can be related to outcome expectations and self-views in ways that make an intended path feel attainable and desirable, which can produce movement in career intentions even when interest is held constant in the theoretical model. In that sense, direct paths from experience to intentions are conceptually permissible when repeated activities crystallize identities, clarify valued outcomes, or reduce perceived barriers to entry [12,14].

Continued usage intentions of AI in coursework is a plausible candidate for such a direct influence. Iterative prompt and feedback cycles can generate mastery experiences and a sense of autonomy, which self-determination theory links to internalization of goals and to stronger self-endorsed intentions. Expectancy–value accounts likewise imply that when students repeatedly experience success with AI on valued tasks, they internalize the perceived utility of the domain and form plans that reflect that utility, even apart from concurrent shifts in interest level. These processes describe a route by which sustained, effective engagement with AI could relate directly to intentions to pursue study or work in business analytics, while still acknowledging that interest remains the more proximal determinant in the canonical SCCT pathway [14,48,49].

The magnitude of any direct link is likely smaller than the interest-mediated pathway emphasized in SCCT. It may also depend on the quality of engagement, since intentions tend to follow productive, goal-congruent activity rather than mere exposure to tools or time on task. That observation aligns with work showing that interest and related motivational outcomes are promoted when learners experience progress on valued goals and see clear relevance of activities to those goals, conditions that also support intention formation when tools reliably help students achieve what they value in their coursework [15].

H4: Continued AI usage intentions will be positively associated with students’ intentions to pursue a career in business analytics.

**Fig 1:**
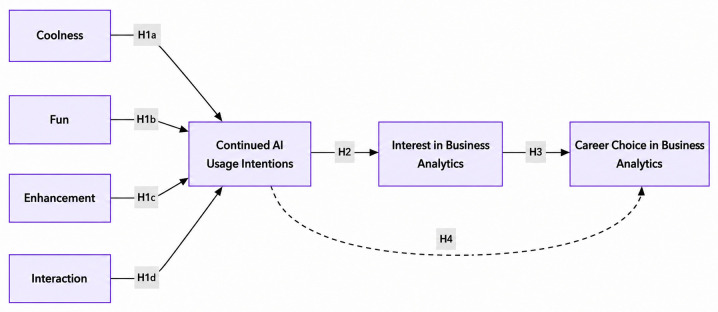
Conceptual model.

Fig 1 presents the conceptual model examined in this study, illustrating the relationships among AI-supported learning perceptions, continued AI usage intentions, academic interest, and career choice intentions.

## Method

### Research design

This study employed a survey-based, cross-sectional quantitative research design to examine the relationships among students’ perceptions of AI tools, continued AI usage intentions, academic interest, and career choice intentions in business analytics. Data were collected via an online questionnaire administered to students enrolled in business-related programs, and the proposed theoretical model was tested using structural equation modeling.

### Data collection

This study was approved by the Institutional Review Board of North Carolina State University (IRB Protocol No. 27465). The research team administered an online Qualtrics survey to students majoring in business-related fields at a large Southeastern U.S. university. All participants provided informed consent prior to participation. A total of 253 students began the survey, of whom 143 provided complete responses and were included in the analyses, resulting in an attrition rate of approximately 43%. Data collection occurred over a four-week period between October 23 and November 18, 2024.

### Participants

The final sample consisted of 143 students. Among them, 58 (40.6%) identified as male and 85 (59.4%) as female. The majority of respondents were between 18 and 24 years old (95.8%, n = 137). A smaller portion of the group fell into older age brackets: four participants (2.8%) were between 25 and 29, while one participant each (0.7%) reported being 30–34 and 35–39 years old.

With regard to racial and ethnic composition, most students identified as White/Caucasian and non-Hispanic (69.2%, n = 99). Other groups represented included Asian/Pacific Islander (18.2%, n = 26), Hispanic/Latino (4.2%, n = 6), Black/African American (2.8%, n = 4), Native American/American Indian (1.4%, n = 2), and Other (4.2%, n = 6).

Educationally, nearly all participants were undergraduates. The largest group was juniors (32.9%, n = 47), followed by sophomores (23.8%, n = 34), seniors (21.7%, n = 31), and freshmen (14.7%, n = 21). A few identified as high school students in 12th grade (4.9%, n = 7) or 10th grade (0.7%, n = 1), while two students (1.4%) selected “Other.” These participants were retained because they were enrolled in business-related or analytics-related learning contexts associated with the institution, such as pre-college, dual-enrollment, or outreach learning opportunities.

Students also reported their family’s socioeconomic status using a five-point scale. Nearly half classified themselves as middle class (46.2%, n = 66), followed by upper-middle class (30.8%, n = 44) and lower-middle class (16.8%, n = 24). Only small percentages were identified as lower class (4.9%, n = 7) or upper class (1.4%, n = 2). Academic standing was assessed through self-reported grades in their most recent business analytics (BA) course or overall GPA from the past year. A large proportion of students indicated high achievement, with 39.9% (n = 57) reporting an A and 25.9% (n = 37) reporting an A–. The remaining distribution included B+ (16.8%, n = 24), B (10.5%, n = 15), and B– (5.6%, n = 8). Only two students reported grades of C+ (0.7%) or C (0.7%).

The sample reflects a relatively specialized population of students enrolled in business analytics–related coursework. At NC State, business analytics is offered as a concentration within broader degree programs and represents a relatively recent curricular development, resulting in comparatively smaller and distributed enrollment sizes.

### Measures

The study employed validated multi-item instruments adapted from prior research, primarily drawing on frameworks of AI-mediated communication and Social Cognitive Career Theory [12,51,52]. Seven constructs were evaluated: coolness, fun, enhancement, interaction, continued AI usage intentions, interest in business analytics (BA), and career choice. All constructs were assessed on a 5-point Likert scale ranging from 1 = strongly disagree to 5 = strongly agree. Descriptive statistics, means, standard deviations, and Cronbach’s alpha coefficients for each construct are reported in Table 1.

**Table 1:** Measurement items.

Construct & Source	Item	Factor Loading
**Coolness** **Wang et al., 2016** M = 3.95, SD = 0.71; α = .84	It is cool.	0.83
	It is distinctive.	0.89
	It is stylish.	0.75
**Enhancement** **Cheng et al., 2022** M = 3.93, SD = 0.75, α = .77	It allows me to freely ask my questions.	0.80
	It allows me to express my insights.	0.69
	It allows me to freely assert my identity.	0.80
**Fun** **Cheng et al., 2022** M = 3.72, SD = 0.83, α = .74	It is fun to explore.	0.87
	It lets me play.	0.71
	I enjoy interacting with the AI tool.	0.69
**Interaction** **Cheng et al., 2022** M = 4.03, SD = 0.63, α = .86	I expect to interact with the AI tool.	0.79
	I can perform a number of tasks via this AI tool.	0.88
	I can specify my needs and preferences on an ongoing basis.	0.85
**Continued AI usage Intentions (Cheng et al., 2022)** M = 4.16, SD = 0.62, α = .85	I am happy with my current experience with AI tools.	0.88
	I would recommend using AI tools to others.	0.86
	I plan to continue using AI tools.	0.74
	I intend to keep using AI tools in the future.	0.75
**Interest in BA** **(Chiu et al., 2023)** M = 4.04, SD = 0.63, α = .78	I would like to collaborate with business analysts.	0.82
	I would like to learn about the latest advancements in business analytics.	0.77
	I would like to practice more skills related to business analytics.	0.66
	I would like to join more curricula related to business analytics.	0.66
**Career-Choice Intentions in BA** **(Chiu et al., 2023)** M = 3.51, SD = 0.80, α = .87	Pursuing a career in business analytics is my dream.	0.81
	Business analytics activities align with my interests and personality.	0.82
	I want to pursue a career in business analytics.	0.82

**Coolness.** Three items adapted from Wang et al. (2016) assessed students’ perceptions of the distinctiveness and stylishness of AI tools. A sample item included “It is distinctive.” Reliability was strong (α = .84; M = 3.95, SD = 0.71).

**Enhancement.** Three items from Cheng et al. (2022) measured whether AI tools enhanced self-expression and identity assertion (e.g., “It allows me to freely ask my questions”). The scale showed acceptable reliability (α = .77; M = 3.93, SD = 0.75).

**Fun.** Three items adapted from Cheng et al. (2022) evaluated the playful and enjoyable aspects of using AI tools. A sample item included “It is fun to explore.” Reliability was acceptable (α = .74; M = 3.72, SD = 0.83).

**Interaction.** Three items from Cheng et al. (2022) measured expectations for active engagement with AI tools (e.g., “I can perform a number of tasks via this AI tool”). Internal consistency was high (α = .86; M = 4.03, SD = 0.63).

**Continued AI usage intentions.** Four items adapted from Cheng et al. (2022) captured satisfaction and behavioral intentions regarding AI use (e.g., “I intend to keep using AI tools in the future”). The construct demonstrated strong reliability (α = .85; M = 4.16, SD = 0.62).

**Interest in Business Analytics.** Four items adapted from Chiu et al. (2023) measured students’ motivation and enthusiasm for BA (e.g., “I would like to learn about the latest advancements in business analytics”). Reliability was satisfactory (α = .78; M = 4.04, SD = 0.63).

**Career-choice intentions in BA.** Three items adapted from Chiu et al. (2023) assessed students’ intention to pursue business analytics as a career (e.g., “Pursuing a career in business analytics is my dream”). Reliability was excellent (α = .87; M = 3.51, SD = 0.80).

**Control variables:** Demographic variables such as gender, income, and ethnicity, and prior AI usage frequency were measured and controlled in the structural model. Participants reported their frequency of using various AI-powered educational tools over the past 12 months. The survey included 10 popular education tools serving different learning purposes: tutoring and content generation (ChatGPT), writing assistance (Grammarly), personalized learning (Khan Academy, Duolingo, DreamBox), quiz generation (Quillionz), and homework help (Socratic). In the analysis, the reported frequency of AI tool use was included as a control variable to account for individual differences in prior exposure to AI technologies, which could be associated with participants’ perceptions, evaluations, or interactions with AI. Responses were rated on a 5-point scale: 1 = *Never*, 2 = *Sometimes*, 3 = *About half of the time*, 4 = *Most of the time*, and 5 = *Always*. (α = .87. M = 1.79, SD = .52). The survey included 10 named AI-powered or AI-supported educational tools, plus an “Other” option. Descriptive statistics also indicated notable variation across different tools. Participants reported relatively higher usage for general-purpose and widely accessible tools (e.g., ChatGPT, Grammarly), compared to lower usage for more specialized or context-dependent platforms (e.g., DreamBox, Edmodo). This suggests that participants’ prior AI experience was selective rather than uniformly limited.

## Results

### Measurement model

The confirmatory factor analysis (CFA) indicated that the measurement model demonstrated an acceptable fit to the data. The chi-square test was significant, χ² = 333.63, df = 201, χ²/df = 1.66, indicating good model parsimony. Additional fit indices supported the adequacy of the model: SRMR = 0.05, RMSEA = 0.06 (90% CI = 0.05–0.08), IFI = 0.94, TLI = 0.92, and CFI = 0.94. Collectively, these indices suggest satisfactory overall model fit [53]. Factor loadings for each construct ranged from.65 to.89 (as shown in Table 1). The reliability and convergent validity of each construct were assessed using composite reliability (CR) and average variance extracted (AVE). All CR values were above 0.81, and all AVE values were above 0.53, confirming excellent reliability and good convergent validity [54,55]. Discriminant validity was confirmed using the Fornell–Larcker criterion, as the square root of each construct’s AVE exceeded the highest inter-construct correlations (as shown in Table 2). Overall, the measurement model demonstrated strong psychometric properties, supporting the validity of the constructs and their use in subsequent structural model analyses.

**Table 2:** Correlations of Key Variables.

Variable	Coolness	Enhancement	Fun	Interaction	Continued AI usage intentions	Interest	Career Choice
**Coolness**	**0.82**	0.53**	0.26**	0.62**	0.54**	0.22**	0.11
**Enhancement**	—	**0.77**	0.48**	0.48**	0.58**	0.19*	0.23**
**Fun**	—	—	**0.76**	0.35**	0.51**	0.17*	0.09
**Interaction**	—	—	—	**0.84**	0.59**	0.14	0.00
**Continued AI usage intentions**	—	—	—	—	**0.81**	0.16	0.05
**Interest**	—	—	—	—	—	**0.73**	0.60**
**Career Choice**	—	—	—	—	—	—	**0.82**

**Notes:** Diagonal = √AVE; *p < 0.05, **p < 0.01

### Results of the structural model

The hypothesized structural model demonstrated good fit with the data: χ²(267) = 424.00, p < .001, χ²/df = 1.59, SRMR = .06, RMSEA = .06 (90% CI = .05–.08), CFI = .92, TLI = .90, IFI = .93, with a sample size of 143. The structural equation model estimated approximately 59 free parameters, including factor loadings, error variances, latent variances, covariances, and structural paths. In addition, an RMSEA-based power analysis was conducted (rmsea₀ = .05, rmsea₁ = .08, α = .05, df = 267). The results indicated high statistical power for the current sample size (N = 143; power = .996) for detecting meaningful deviations from close model fit. These results suggest that the model demonstrates adequate sensitivity to detect overall model misfit at the current sample size. A Harman’s single-factor test was also conducted to assess common method bias. The first factor accounted for 33.87% of the total variance, which is below the commonly used threshold of 50%, suggesting that common method bias is unlikely to fully explain the observed relationships.

### Direct effects

**Fig 2:**
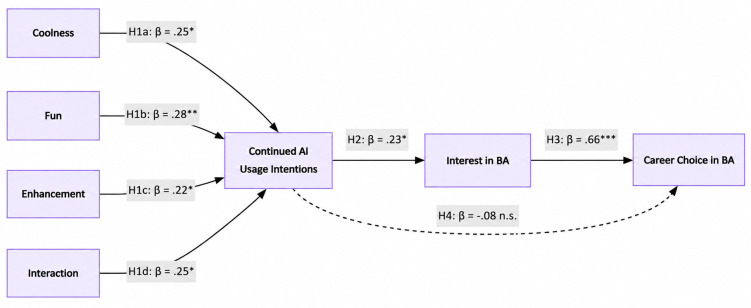
Structural model with results.

As shown in Fig 2, H1a through H1d examined the antecedents of continued AI usage intentions. The results revealed that coolness (β = .25, p = .027), fun (β = .28, p = .003), enhancement (β = .22, p = .045), and interaction (β = .25, p = .028) were each positively and significantly associated with students’ intentions to continue using AI tools. These findings highlight that aesthetic appeal, enjoyable experiences, perceived agency, and active engagement with AI tools are important predictors of ongoing usage intentions.

Supporting H2, continued AI usage intentions were positively related to interest in BA (β = .23, p = .016), suggesting that continued AI usage intentions are associated with greater motivation and enthusiasm for business analytics.

In examining H3 and H4, Interest in BA was strongly associated with career choice in BA (β = .66, p < .001), supporting H3. The direct effect of continued AI usage intentions on career choice was not significant (β = −.08, p = .318), thus H4 was not supported. This indicates that interest acts as the primary mediator between continued AI usage intentions and career intentions in business analytics.

### Indirect effects

Mediation analyses using bias-corrected bootstrapping (5,000 samples) indicated that academic interest statistically mediated the relationship between continued AI usage intentions and career choice intentions. The indirect effect from continued AI usage intentions to career choice through academic interest was positive and statistically significant (β = .19, p = .013, BC 95% CI [.040,.390]).

In contrast, none of the indirect effects from the antecedent variables (coolness, fun, enhancement, and interaction) to academic interest through continued AI usage intentions were statistically significant, as all corresponding confidence intervals included zero. Additionally, the direct pathway from continued AI usage intentions to career choice was not significant, further highlighting the role of academic interest as the primary mechanism linking usage intention to career-related outcomes.

## Discussion

This study reflects current instructional practices in U.S. business analytics education, where such programs are often offered as concentrations rather than standalone majors, resulting in smaller and more specialized student populations. This study examined how students’ engagement with AI-supported learning environments is associated with continued AI usage intentions in coursework and how such sustained usage intentions are related to academic interest and career-related intentions in business analytics education. Positioned at the intersection of post-adoption technology research and educational perspectives on learning experience design, the findings suggest that both functional and experiential qualities of AI-supported tools are critical for sustaining engagement in authentic instructional contexts. Specifically, perceived enhancement, interaction, fun, and coolness each significantly predicted students’ continued usage intentions with AI tools as part of their regular coursework. However, the mediation analysis did not support indirect effects from these antecedent variables to academic interest through continued AI usage intentions, suggesting that these factors primarily operate at the level of sustaining engagement rather than directly shaping interest development. These results suggest that sustained AI use in education is driven not by utility alone, but by a combination of effectiveness, engagement, and experiential value embedded within course-based learning activities.

An important contextual factor is the relatively low overall level of prior AI usage (M = 1.79). However, this aggregate measure masks variation across tools, with higher usage reported for general-purpose applications (e.g., ChatGPT, Grammarly) and lower usage for more specialized platforms. This suggests that students’ AI experience is selective rather than uniformly limited. This pattern reflects an early and uneven stage of AI adoption, where perceptions are formed based on partial experience. While this context may be related to how students evaluate AI-supported learning, it also enhances the relevance of the study by capturing user perceptions during early adoption. Caution is warranted when generalizing to more experienced populations. Consistent with post-adoption research, perceived usefulness and satisfaction remain central drivers of continued usage intentions once initial exposure has passed, while experiential qualities such as enjoyment and involvement further support sustained use in mixed utilitarian–hedonic systems [7,11,23]. From an educational perspective, the findings align with research emphasizing that responsiveness, perceived control, and interactive features are related to learners’ experiences during repeated engagement with digital tools in real learning environments [24,26]. In AI-supported coursework, interaction and interface cues appear to be related to how students experience learning tasks over time, reinforcing continued usage intentions as part of routine academic practice rather than episodic experimentation.

The findings further link continued AI usage intentions to motivational and career development processes central to educational research. Interest tends to develop when repeated learning experiences reinforce perceptions of competence and value, a pattern consistent with expectancy–value theory and self-determination theory, both of which emphasize competence and autonomy as key supports for intrinsic motivation and interest development [16,48,49]. Social Cognitive Career Theory (SCCT) situates interest as a proximal antecedent of choice goals and career-related intentions, organizing these processes around learning experiences that are related to self-efficacy beliefs and outcome expectations [12,14]. Meta-analytic evidence indicates that these pathways are robust across domains, including science and technology-related fields [13,50].

Within this framework, the study makes three contributions to research on technology, knowledge, and learning. First, it integrates design-level perceptions of AI-supported learning environments with motivational and career-related outcomes, demonstrating how the sustained usage intention is associated with academic interest development, which in turn is linked to career-related intentions, while no indirect effects from design-related antecedents to academic interest were supported.

Second, the findings clarify why simple usage metrics are insufficient for understanding longer-term educational outcomes: it is the motivational quality of engagement during learning activities, rather than frequency alone, that predicts interest and intention formation. Third, the study extends continuance research by highlighting socio-aesthetic value, captured through perceived coolness, as a complementary factor alongside usefulness and enjoyment. This value appears most consequential when embedded in ongoing, productive learning experiences rather than isolated novelty effects [27,30].

### Antecedents of Continued AI Usage Intentions in Coursework

The results support a dual-pathway account in which functional enhancement and experiential rewards jointly sustain AI use in educational settings. Enhancement reflects students’ perceptions that AI tools support inquiry, self-expression, and meaningful engagement with learning activities, thereby reinforcing intentions for continued use, while interaction, fun, and coolness capture experiential and affective dimensions that support engagement during repeated use. Interaction, characterized by user control, contingent responsiveness, and two-way exchange, appears particularly relevant in AI-supported learning, where iterative prompt–feedback cycles can reinforce perceived efficacy and satisfaction during authentic academic tasks [24,25].

Socio-aesthetic value further complements these drivers. Perceived coolness reflects distinctiveness and appropriate novelty that signal social and symbolic appeal, which may elevate students’ willingness to continue using AI tools as part of their academic identity. Importantly, the findings suggest that coolness operates most effectively when paired with visible progress and a sense of control, rather than as a standalone novelty effect. As routines form and novelty declines, design features that support meaningful progress and learner agency become increasingly central to sustained usage intentions over the course of a semester [8].

### From Continued Usage Intentions to Academic Interest and Career Intentions

Continued AI usage intentions in coursework were positively associated with students’ academic interest in business analytics, which is consistent with the view that sustained usage intentions may function as a motivational learning context. Interest development is understood as a progression from triggered situational interest to maintained situational interest and, ultimately, emerging individual interest, with repeated effective experiences strengthening competence beliefs and perceived value [16,48]. AI-supported activities that allow students to feel effective, autonomous, and goal-directed may therefore be associated with stronger academic interest. SCCT provides a complementary explanation for how interest development connects to career-related intentions. Within this framework, hands-on learning experiences are related to self-efficacy and outcome expectations, which foster interest and guide choice goals [12,14]. The present findings align with evidence that the interest–choice relationship is robust across educational domains and underscore that it is the quality of engagement, rather than mere exposure, that supports this process [13,15]. By conceptualizing continued AI usage intentions as reflecting an ongoing learning-related orientation, the study demonstrates how AI-supported instruction is associated with academic interest, which in turn is related to career intentions in business analytics education.

Notably, the direct relationship between continued AI usage intentions and career choice intentions was not statistically significant and was negative in direction (β = −0.08). This finding suggests that intention to continue using AI tools in coursework does not directly translate into career-related intentions in this context. Instead, the results indicate that academic interest serves as the primary mechanism linking AI-supported learning experiences to career orientation. This pattern is consistent with Social Cognitive Career Theory, which positions interest as a proximal antecedent of career-related goals rather than a direct outcome of learning behaviors alone [12,14]. It also suggests that continued usage intentions with AI tools may need to be meaningfully connected to disciplinary interest before influencing students’ career intentions.

This study advances theory at the intersection of education and information technologies by linking post-adoption continuance research with career development perspectives in an AI-enabled classroom context. The findings suggest how motivational factors that sustain students’ continued usage intentions of AI-supported learning tools contribute to the quality of learning experiences that foster academic interest, which Social Cognitive Career Theory (SCCT) identifies as a proximal antecedent of career-relevant intentions. By integrating continuance antecedents with expectancy–value and self-determination processes, the interpretation clarifies how perceptions of usefulness, enjoyment, and control during coursework can initiate and sustain interest development and, in turn, support intention formation related to future educational and career pathways [7,11,12,14,23,48,49].

A second theoretical contribution concerns the role of interface affordances in post-adoption educational contexts. Rather than treating interaction as a channel feature alone, the findings conceptualize interaction as an experiential construct capturing perceived responsiveness, user control, and two-way exchange during repeated engagement with AI tools. These elements contribute to satisfaction and perceived usefulness during iterative prompt–feedback cycles. While related interface characteristics such as modality, agency, and navigability have been discussed in prior research as heuristic cues that shape users’ rapid evaluations of digital systems, they represent conceptually distinct dimensions and were not operationalized as equivalent to interactivity in the present study [24–26].

A third implication highlights the theoretical value of socio-aesthetic perceptions in sustaining educational technology use. Perceived coolness extends the conventional focus on usefulness and enjoyment by capturing the social and symbolic appeal of AI tools when distinctiveness and appropriate novelty are present. Importantly, coolness is positioned as complementary to, rather than a substitute for, functional and experiential rewards. This framing helps explain when socio-aesthetic value is most likely to matter for sustained usage intentions in educational settings—namely, when tool design continues to support visible progress and a sense of learner control as novelty diminishes [8,27,30].

The study also contributes to construct clarity in research on AI-supported learning. Continuance is operationalized as students’ intentions to continue using AI tools for routine coursework, rather than as behavioral frequency or time-on-task measures. Academic interest is distinguished from short-lived curiosity by its relative stability and its role in guiding subsequent learning and career-related choices. Career intentions are conceptualized as plans rather than enacted behavior. These distinctions align measurement choices with theoretical definitions and reduce conceptual drift across continuance, motivation, and career development literature [14,16].

Finally, the findings point to boundary conditions that warrant explicit theorization in future research on education and information technologies. Task-technology fit, classroom norms, and institutional expectations may condition how motivational perceptions translate into continued usage intentions and downstream motivational outcomes, while habit formation can stabilize behavior as novelty declines. Together, these considerations suggest a moderated mediation perspective in which the strength of relationships among design perceptions, continuance, interest, and intentions varies by instructional context, transparency, and the reliability of AI outputs [8,56].

### Practical implications

The findings of this study offer preliminary insights for AI-supported learning in business analytics education, although they should be interpreted within the context of a single institution and a cross-sectional design. Rather than prescribing specific instructional approaches, the results highlight several considerations for how AI-supported learning may be structured to support student engagement and motivation.

At the instructional level, the findings suggest that integrating AI tools into routine learning activities may be associated with more sustained student engagement than using them as occasional demonstrations. When AI-supported tasks are aligned with course objectives and allow for iterative interaction, students may be more likely to perceive these tools as useful and relevant to their learning. Instructional supports related to AI use may help students connect continued AI usage intentions with academic interest. These patterns are consistent with prior research on engagement and motivation in technology-supported learning environments, as well as with the observed relationship between continued AI usage intentions and academic interest in the present study.

At the curriculum level, the findings suggest that distributing AI-supported learning activities across courses, rather than limiting them to isolated experiences, may provide students with repeated opportunities to engage with AI in meaningful ways. Such repeated exposure may support the development of academic interest over time, particularly when AI use is aligned with core disciplinary skills such as data interpretation, communication, and decision-making.

At the program level, the findings suggest that AI-supported learning may be associated with students’ emerging academic and career orientations when connections between coursework and career pathways are made more explicit. For example, linking AI-supported assignments to internships, projects, or professional competencies may help students interpret their learning experiences in relation to future goals. This interpretation aligns with prior research on expectancy–value processes and career development, although the current study does not directly test program-level interventions or long-term outcomes.

Overall, these implications should be interpreted as context-dependent and preliminary. Given the study’s design, future research is needed to examine how AI-supported learning influences student engagement, interest, and career development across different institutional contexts and over time.

### Limitations & Future research

This study is subject to several limitations that suggest directions for future research. First, the data were drawn from a single program at one institution, and findings may reflect local curriculum design, classroom norms, or prior exposure to AI tools. In addition, a small number of participants identified as high school students enrolled in pre-college or outreach learning contexts associated with the institution. Although they represented a small proportion of the sample, their inclusion may introduce heterogeneity in educational background and experience. Furthermore, the relatively high attrition rate may introduce response bias, as participants who completed the survey may differ systematically from those who did not. The sample size (N = 143) is also modest relative to the complexity of the structural equation model, which includes multiple latent constructs and estimated parameters. Although the model demonstrated acceptable fit indices and RMSEA-based power was high, the sample size may still limit the stability of parameter estimates and the generalizability of the findings. Future research should examine more clearly defined undergraduate populations and broader institutional contexts to assess generalizability.

Second, the cross-sectional design limits conclusions about temporal ordering among continued AI usage intentions, academic interest, and career intentions. Longitudinal, multi-wave designs across a semester could better capture motivational change as novelty fades and habits form. Third, continued AI usage was measured as intention rather than observed behavior, which may limit the interpretation of actual engagement. Future research may combine self-reports with behavioral indicators, such as assignment-linked AI usage or system logs, to provide a more comprehensive assessment of engagement.

Finally, because all variables were measured using self-reported data collected in a single survey session, common method bias may have influenced the observed relationships. A Harman’s single-factor test indicated that the first factor accounted for 33.87% of the total variance, suggesting that common method bias is unlikely to fully explain the findings; however, this approach has limitations. Future research should incorporate both procedural and statistical remedies, such as multi-source data or temporal separation, to further mitigate this concern [57]. Given the rapid evolution of generative AI tools, future studies should also report tool versions and features to support comparison across contexts and over time.

## Conclusion

This study examined how motivational perceptions of AI-supported learning tools relate to continued usage intentions in coursework and how sustained usage intentions connect to academic interest and career-related intentions in business analytics education. Integrating post-adoption continuance research with motivation and career development theory, the findings show that continued AI usage intentions reflect both functional enhancement and experiential rewards, and that academic interest serves as the most proximal link between classroom engagement and career-relevant planning. Conceptually, the study clarifies why learning experiences that build competence and perceived value are more informative for long-term educational outcomes than simple exposure or frequency of use, while sharpening distinctions among continuance intention, enduring interest, and career intentions. Practically, the results highlight the importance of embedding AI in authentic tasks, scaffolding effective interaction and learner choice, and ensuring ethical and transparent use to support sustained usage intentions and inclusive motivation. Although based on a single instructional context, the study points to clear directions for future research, including replication across settings, longitudinal designs, and behavioral measures, and underscores an actionable pathway through which AI-supported coursework can foster interest development and emerging career thinking in higher education. These findings should be interpreted within the context of the study setting. The sample was drawn from a single institution in the Southeastern United States, and the results may reflect local curricular structures, institutional characteristics, and student populations. Therefore, the generalizability of the findings to other institutions, regions, or disciplinary contexts may be limited. Future research should replicate this model across diverse educational settings to assess the robustness and broader applicability of the findings.
